# New Models to Reduce the Health Risks of Informal WEEE Recyclers in MTN Phone Village, Rumukurushi, Port Harcourt, Nigeria

**DOI:** 10.3390/toxics10020084

**Published:** 2022-02-12

**Authors:** Ogechukwu Okwu, Andrew Hursthouse, Evi Viza, Linus Idoko

**Affiliations:** 1 School of Computing, Engineering & Physical Sciences, University of the West of Scotland, Paisley PA1 2BE, UK; andrew.hursthouse@uws.ac.uk (A.H.); evi.viza@uws.ac.uk (E.V.); 2 Department of Electronic & Computer Engineering, Veritas University, Bwari, Abuja 900001, Nigeria; idokol@veritas.edu.ng

**Keywords:** informal recycling, Port Harcourt, WEEE management, hazard, risk assessment

## Abstract

Waste electrical and electronic equipment (WEEE) management in Port Harcourt, an oil-producing city in Nigeria, has become an environmental challenge for the location. WEEE recycling is predominantly managed by informal recyclers, who lack the skills to perform risk-free recycling, hence raising health risks to individuals in associated communities and degrading the environment. Formal recycling, which embraces the best practices for effective WEEE management, is faced with several limitations, such as a lack of detailed guidelines on waste recycling, reuse, and final disposal techniques, with no opportunities for landfilling. A qualitative approach was adopted for this study. Data were gathered via questionnaires and analysed graphically. A background literature review of the assessment of informal recycling methods and associated challenges was performed. Hence, a new concept for the local management of WEEE processing was introduced. This concept limits the role of informal recyclers to WEEE collection. In this case, informal recyclers are paid for WEEE collection; they no longer engage in further WEEE processing. The results show that 48% and 40% agree to partner and collaborate with government agencies, respectively. Conversely, 52% and 40% agree and strongly agree, respectively, to limit their activities to WEEE collection only if the government is willing to pay for the services.

## 1. Introduction

Port Harcourt, an oil-producing city in Nigeria, experiences a massive number of individuals arriving in search of financial benefits from the oil and gas industries. Because of this ever-rising number of inhabitants there is an increase in the consumption of materials as well as the generation of waste electronic and electrical equipment (WEEE) and other environmental contaminants. The quantity of WEEE produced has been steadily increasing [1]. Furthermore, according to the same study this is largely a result of the increasing need for information and communications technology as well as enhanced technology in some parts of the world, resulting in the dumping of “old and discarded” electronic and electrical equipment to countries such as Nigeria, where such items are still useful. These items are usually obsolete or close to obsolescence before they are transported to poor nations, which tends to increase the quantity of generated WEEE. According to [2], WEEE, which is among the biggest and constantly increasing streams of global waste, has become an issue of serious concern due to its associated challenges. The problem created by WEEE appears to be twofold. First and foremost, it is among the most rapidly increasing waste streams globally, presenting a potential threat to humans, animals, and the environment as a result of its mismanagement. Secondly, as a result of its composition, which is complex, WEEE is also one of the most difficult waste streams to manage effectively [3]. In Port Harcourt, a city in the south–south geopolitical zone of Nigeria, the WEEE collectors do not practice formal recycling methods. The system in practice is the informal recycling method, carried out primarily at the dumpsite, where they engage in the sorting of WEEE as well as further recycling [4]. This begs the following question: how can we tackle the challenges associated with informal recycling?

Ohajinwa et al. [5] put forward the notion that informal recyclers engage in several activities, which include the recycling of WEEE. Their services are usually provided at a low cost, but the working procedure used is not safe; hence, their health is at risk from exposure to substances within the materials and from the operations used in recycling, such as burning to remove plastics. In a related study [4], WEEE and other waste are usually gathered unsorted at locations in Port Harcourt, such as Igwuruta, Elelenwo, and Abuloma. WEEE serves as a source of raw materials, but during the informal recycling process the estimated recovery of useful metals is only 25% [6,7].

In a study on WEEE generation by Mihai et al. [8] this growing waste stream and its inappropriate handling generate serious pollution- and health-related challenges. This can result from the dismantling of WEEE being carried out under “poor conditions”. The dumping of WEEE in Africa has been significant for a long period, and its recycling is usually carried out by poorly educated individuals, under temporary conditions, with no infrastructure, which leads to exposure to harmful substances. Due to a lack of organised collection centres in Nigeria WEEE is dumped together with waste from hospitals and other sources in the community, which causes the situation to be more complex. In some communities WEEE is dumped in bodies of water and on open fields, resulting in direct environmental pollution. Items considered to be of no use economically are usually burnt regularly to reduce their quantity or directly deposited on open fields or in water bodies. The indiscriminate discarding of WEEE has resulted in an increase in polybrominated diphenyl ether and polychlorinated biphenyl concentration in individuals. Fluorinated biphenyls and analogues are also toxic pollutants emitted due to the indiscriminate dumping of WEEE [9,10,11,12,13,14]. 

Electronic appliances such as televisions and computer monitors contain potentially harmful elements and compounds, which are evident during disposal or recycling. The majority of those who make use of them are not aware of the associated risks of using them frequently [15]. The landfills, as well as unauthorised dumpsites, appear to be the terminal point of the majority of the gathered WEEE [16]. Harmful gases are usually released into the environment during the burning of WEEE [12,17]. The disposal and recycling of cathode ray tubes (CRTs) have the potential to expose life to health risks due to the presence of lead in CRT funnel glass (at levels up to 22%). In addition to lead, CRTs consist of fluorescent powders, barium, and cadmium, all of which are toxic. The harmful elements contained in CRTs are easily absorbed in the soil over time and are consequently passed to humans and animals [18,19,20,21]. The presence of devices that contain mercury causes soil pollution and contributes to increased health risks. Mercury is more poisonous than lead and cadmium, and it causes loss of hearing and vision as well as developmental delays [22,23,24]. The tissue and surface of locally grown fresh vegetables can easily become contaminated when exposed to residues from waste processing directly deposited on fields and in water. Skin contact and drinking water have also been recognised as significant routes of exposure to toxic substances [25,26,27].

**Figure 1:**
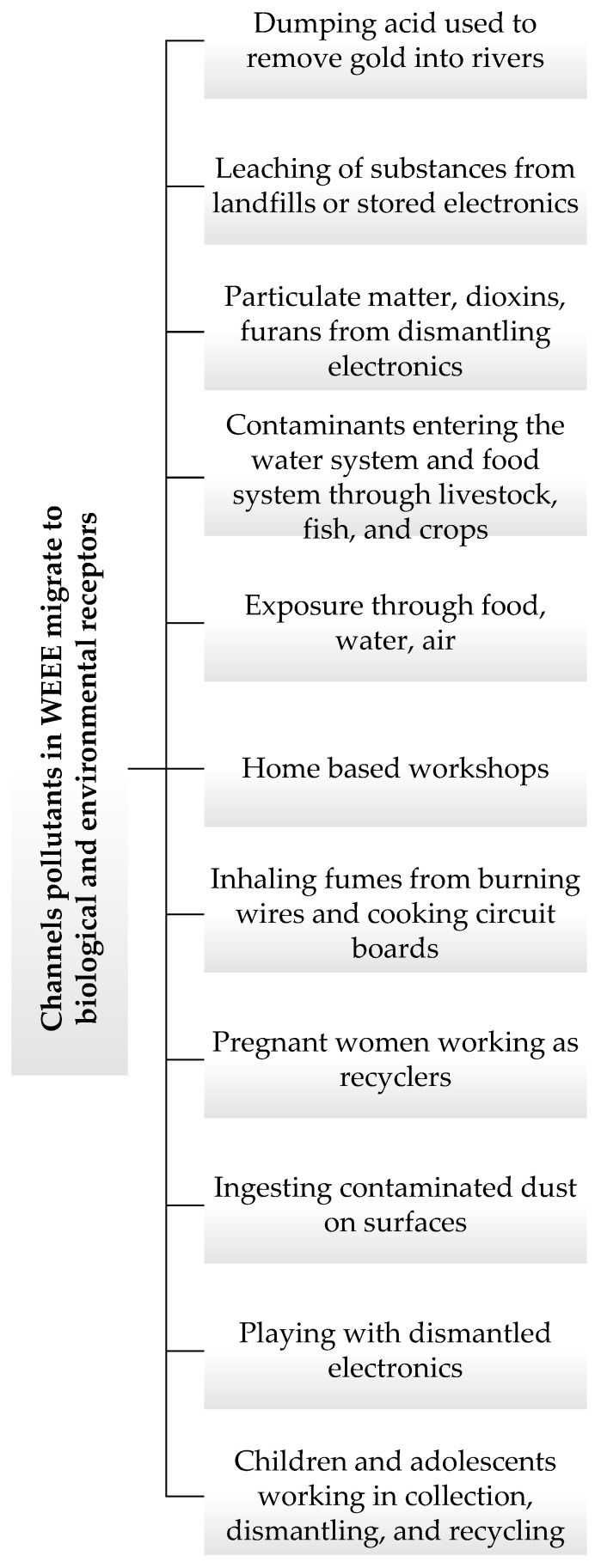
Channels pollutants in WEEE migrate to biological and environmental receptors.

Unprofessional WEEE burning and dismantling methods contribute significantly to the pollution of air. This can result in secondary exposure, as some of the pollutants are able to travel over a long distance to other locations from the recycling sites [28]. The soil, as well as the crops grown in the WEEE dumpsites, are usually exposed to a high concentration of metals, such as lead, zinc, copper, etc. The accumulation and uptake of them by plants constitute the major entry channel for metals, which are toxic in animal and human food. Examples of such toxic metals are metalloids such as lead, mercury, cadmium, selenium, and arsenic. Crops that grow in the metal-polluted locations usually show a reduction in growth, reduced biomass production, accumulation of metal, and alteration of metabolism. It is common practice for locals to grow crops around and within WEEE dumpsite areas, as they believe the land is fertile and that crops will grow well. Research has demonstrated that many locations contain high levels of potentially toxic elements [29,30,31,32]. The pollutants in WEEE migrate towards biological and environmental receptors via several pathways [33]; the most relevant for informal recycling activities in Nigeria are summarised in Figure 1.

In recent research conducted by George et al. [34] the increasing quantity of WEEE is shown to be associated with several factors, which include technological improvement, the availability of assorted electronic and electrical devices for sale, the reduction in prices of electronic and electrical devices, and the growing population, fuelling an extremely “high demand” for them. WEEE is a potential source of rare metals and useful plastics, helpful in reducing the pressure on mineral resources, the exploitation of natural resources, as well as the environmental and cost implications of mining. Several methods are adopted to extract metals. For example, studies have shown that the copper present in printed circuit boards (PCBs) can be extracted with the use of a mixture of aqueous acid and supercritical CO_2_. Studies have also shown that rare earth elements can be recovered via electrochemical recovery. In a recent study, important metals, such as copper, silver, and gold, were recovered from the printed circuit boards of computers that were no longer in use via physical separation, after which a leaching technique was applied. In addition, copper can be directly separated and recovered from waste PCBs via slurry electrolysis carried out with an acidic system. Product designs that are environmentally friendly have been encouraged in the European Union via legislation to support the recovery of valuable materials from WEEE [35,36,37,38,39,40,41].

In [42] “the recovery and treatment of WEEE” serve as an alternative source of important elements, for example, copper, gold, etc. However, the indiscriminate disposal of WEEE and the challenges that come with the inappropriate disassembling and management of WEEE, observed in many developing countries, put pressure on the environment and human health [43].

**Figure 2:**
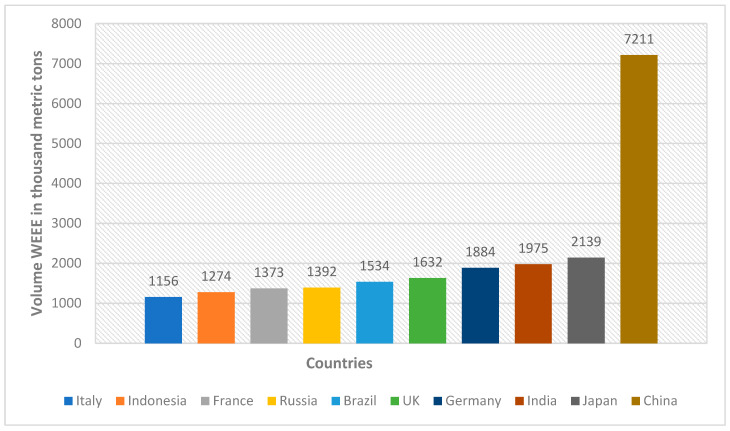
Global generation of WEEE in 2016.

An assessment by Khan et al. [44] indicates that most of the WEEE that is globally generated, amounting to at least 40 million tonnes per year, finds its way to developing countries. Nigeria now appears as one of the locations receiving WEEE from Asia, the US, and Europe [45]. A study by Ferronato and Torretta [43] put forward the notion that developed countries have seen the export of WEEE to Asia and Africa as a preferred option compared to developing their own national recycling infrastructure, encouraging innovative design that limits toxic material use, or switching to cleaner sustainable technologies. In [46] it was observed that the amount of WEEE generated worldwide amounted to approximately “50 million metric tonnes” in 2018. WEEE production is the direct consequence of the manufacturing and use of electronic devices driven by the multipurpose nature of information and communications technologies (ICTs). This increased consumption and comparatively short life of electronics result in a build-up of WEEE, which poses problems at all levels. The hazardous materials in WEEE pose problems for the government in terms of their management [47] and effect on the environment [48]. The estimated quantity of WEEE produced worldwide in 2018 increased to 49.8 metric tons [46]. The major generators of WEEE as of 2014 identified in the same study by Adeola [46], and that of Tiseo [49], are shown in Table 1 and Figure 2, respectively. The statistics show that the United States tops the list in WEEE generated in 2014, while China tops the list in WEEE generated in 2016.

The generation of WEEE and its collection in different continents is identified in Baldé et al. [50] and is summarised in Table 2.

Baldé et al. [50] reported that, in the year 2016, of the bulk of WEEE gathered in Asia, which amounts to 18.2 million metric tons (Mt) (i.e., 4.2 kg/inhabitant), only about 2.7 Mt was recorded for collection and will be recycled. Oceania produced the highest amount, 17.3 kg/inhabitant, and had only a 6% rate of collection and recycling; however, it appears to produce the smallest amount of WEEE in 2016: 0.7 Mt. The statistics for Europe, Asia, the Americas, and Africa are also shown in Table 2. These statistics imply that a large proportion of the WEEE that is collected and recycled is not documented.

We focus on the need to eliminate or reduce the role of recyclers in the informal sector, in particular, to reduce health risks from the management of WEEE in Rumukurushi, in Port Harcourt, Rivers State, Nigeria. This is applicable to similar situations in other cities.

### 1.1. A Review of the Literature on WEEE Management

Several studies have focused on improving the informal management of WEEE. Ogbuanya and Afeez [51] (p. 90) propose that the WEEE approaches applied in the workshops of “electrical/electronic technicians” can be advanced. Data collection was performed using questionnaires administered to 54 “public health” senior staff as well as 87 engineering lecturers. The data analysis was achieved using “percentage, mean, and standard deviation white *t*-test and ANOVA”. The study findings reveal that providing a “recycling site” as well as introducing and applying regulatory policies are the major approaches required for a more effective management of WEEE. However, the study did not directly address the challenges associated with informal recycling but only attempted to identify indicators that may be of help. A study on effective methods that can help to proffer solutions for WEEE management would be valuable.

Arya et al. [52] investigate the degree of understanding of informal recyclers and users (individuals, organisations, and companies) with regard to the hazards created by WEEE. The study collected data via questionnaires administered to three different WEEE stakeholder groups, namely organisations, individuals, and those who use WEEE recycling services. The composition of those who responded to the questionnaires was 10 persons engaged in WEEE recycling, 25 users of electrical and electronic appliances, and 35 organisations considered as users. The study outcomes revealed that the individuals and organisational users lack an understanding of the problems associated with WEEE management. This includes legislation for WEEE management and proper channels for the collection of WEEE. Recyclers in the informal sector were not aware of the risks associated with WEEE and subsequently engaged in unsafe disposal methods. The study did not address the challenges associated with informal recycling, because it primarily focused on the degree of understanding of the informal recyclers and users. The study identified health and safety training as an avenue to increase awareness.

George et al. [34] studied WEEE management with households as their main focus, using an assessment of the electrical and electronic device composition in the apartments of the people in the area of study as well as the way in which WEEE was managed. Data collection for the study was achieved using questionnaires. The study outcomes revealed that the electronic and electrical appliances that appear the most in the area are phones. Furthermore, the study revealed that the selected area adopted an unsustainable system of managing WEEE. Study participants did not recycle WEEE, but simply kept them inside their homes or deposited them directly into the general waste stream. The study did not address the challenges associated with informal recycling because no evaluation was conducted on the activities of informal recyclers; instead, recommendations were offered. Introducing an approach with the potential to provide an effective recycling solution may be helpful in the informal sector.

Ndunda and Ambole [53] (p. 73) tackled the problems introduced by the informal method of WEEE recycling via the creation of “a product-service system” for its management. Supporters of a move, from product provision to “provision of systems of products and services” were developed alongside stakeholders’ support, in order for WEEE to be managed efficiently. Dematerialization technique was applied in the study by making use of the effort of stakeholders’ collaboration, this hinders the informal recyclers or consumers from having possession of equipment after EOL. The outcome indicates that the collaborative efforts of those involved determine the success of the technique. How has the study addressed the challenges associated with informal recycling? The study was unable to address the problem because dematerialisation can only be attained through structural and technological changes in the area, which have yet to be implemented. Adopting an approach applicable to developing nations would be more useful.

In Aidonis et al. [54] a methodology was created to recognise an optimal management scheme for WEEE in order to find another means of integrating WEEE. The study made use of a binary linear programming model to improve the effectiveness of nine options to manage WEEE. Consideration was given to 12 performance criteria, including environmental, financial, social, and technical considerations. The study outcome revealed that the best approaches were exporting WEEE residue and mechanically recycling WEEE. The outcome did not address the issues associated with informal recycling because the technique proposed does not have the potential to tackle the negative repercussions of informal recycling. A technique that has the potential to minimise informal recycling may be more useful.

In Mihai et al. [8] statistical information on waste and “thematic cartography” was used to disclose how WEEE moves from one geographical location to another. The approaches utilised to manage WEEE in numerous locations were examined, for instance, in Europe, North America, South America, Oceania, Africa, etc. The findings indicate that inadequate infrastructure leads to the poor management of WEEE in Africa. The findings did not address the issues associated with informal recycling, as the study focused on elements of the improper management of WEEE. Identifying a useful method that can tackle the challenges facing recycling in the informal sector may be helpful.

### 1.2. Research Gap

It is therefore clear that no major improvements have been achieved to reduce the activities of informal recyclers in Nigeria and other developing countries where WEEE management is predominantly managed by the informal sector. Our study addresses this issue by assessing models for the continued participation of recyclers in the informal sector in the collection of WEEE.

### 1.3. WEEE Status in Nigeria

Goel [55] (p. 8) reports that, as a result of the lack of proper waste management infrastructure and systems in Nigeria, the dumping of WEEE is usually carried out “alongside other municipal waste”; hence, the level of health and environmental risk awareness is “generally low”. The same study explains that the lack of appropriate mechanisms in Nigeria for WEEE “disposal” contributes immensely to the poor knowledge of WEEE disposal and the associated health risks. The inadequate rules and regulations, as well as the poor implementation of laws on sanitation, have created an enabling environment for informal recycling in some parts of Nigeria [56]. The task performed by informal WEEE recyclers is significant and includes the collection of WEEE from streets for recovery, recycling, sorting, and disposal [57]. The job of the informal recycler entails material isolation, dismantling via the use of manual techniques, circuit board heating, the recovery of metal using poisonous acid, and disposal at an open dump [5].

Informal recyclers, also referred to as “scavengers”, usually lack formal skills and are unregistered [58]. A total of 277,000 tonnes of WEEE was generated in Nigeria in 2016. This puts Nigeria in the third position in the hierarchy of WEEE generators on the African continent, with the amount of WEEE expected to increase. No non-governmental or institutional organisation collects or updates data on WEEE, which would serve as a source of valuable information to support policymaking or government action. In addition, a large quantity of WEEE enters the country “from abroad” [50,55]. Some locations in Nigeria, such as Lagos, stockpile WEEE, awaiting the provision of a means to recycle it [45]. The challenges associated with the management of WEEE in Nigeria and other developing countries are further amplified due to the absence of comprehensive and reliable data [59,60].

The problems associated with WEEE management in Africa are well-known, but it still lacks the appropriate infrastructure, evidenced by the absence of appropriate regulatory protocols and enforcement [61]. In the informal sector WEEE is recycled improperly, and the process exposes the community as well as the environment to risk. In general, workers engaged in the informal sector are not aware of the risk associated with potentially toxic and hazardous substances contained in WEEE. Hence, their exposure to these substances can result in severe health challenges. The processing and discharge of toxic products from WEEE are typical of the informal sector. Recycling in the informal sector is carried out without the use of technology and proper protection; thus, individuals incur great risk when processing WEEE [62,63,64]. Developing countries, e.g., Nigeria, lack the infrastructure required for effective WEEE management [10,65].

Adequate awareness and sound understanding of WEEE, which are lacking in recyclers in the informal sector, are necessary to reduce risks to health as well as ineffective disposal, recycling, and reuse. Informal recyclers are usually not registered with the appropriate authorities in their location, and their services are thus illegal [66,67,68].

There is a challenge with the adoption of the extended producer responsibility in several parts of Africa, for example, Nigeria [10,69]. WEEE constitutes a great deal of waste material, both hazardous and non-hazardous. Informal WEEE recycling exposes the recyclers and the neighbouring environment to polybrominated biphenyls, chromium, mercury, cadmium, and lead. This can affect the liver, kidneys, nervous system, and brain. WEEE, which is usually present in municipal solid waste, can give rise to severe health challenges and environmental pollution from the facilities used for incineration and pulverising/disassembling, as well as from sanitary landfills and unauthorised dumping sites [69,70,71,72].

Some of the elements in WEEE that are capable of causing risks to human health and the environment are identified in [73] and shown in Table 3.

### 1.4. Recycling Methods in Nigeria

The two types of recycling methods are those practiced by informal recyclers, also known as scavengers, and those practiced by formal recyclers. Both sectors have a target, which is the management of WEEE in a manner that is profitable and sufficient to ensure that waste is not diverted into the major public waste stream and landfills [74]. Operations common to both informal and formal recyclers, as specified in [75], are the collection, dismantling via the use of manual techniques, recycling, reconditioning, and extraction of cables, metals, plastics, and “printed circuit boards” present in WEEE. In most of the countries referred to as developing, the activities of the informal recycler commence from the moment WEEE is collected and end at the final stage, depending on the available options [76]. The collection as well as the recycling of WEEE in Nigeria is primarily carried out by extremely poor rural Nigerians [58]. The informal recyclers carry out their recycling duties in workshops, which are usually small or in the open air [77].

Countries that are industrialised, such as the US, UK, Sweden, Finland, and Germany, which usually have and maintain “stricter environmental laws and regulations”, practice formal WEEE recycling [74]. Meanwhile, informal WEEE recycling is carried out in countries such as Nigeria, India, Pakistan, Ghana, and China, where environmental laws and regulations are not strictly enforced. Some studies have suggested that informal recyclers make use of common “extraction tools” and systems such as mallets, hammers, screwdrivers, chemical leaching, and open burning, in contrast to formal recyclers who carry out their activities under controlled conditions and with standard equipment. Some of the differences between the informal method of recycling WEEE in Port Harcourt, Nigeria, and the formal methods practiced in other countries, for example, Mexico, are shown in Table 4 [75].

The informal waste management system is the dominant method in Nigeria; this is because formal waste management is faced with several challenges, as specified in [57], including:

- Endless “political interference”.
- An absence of the required facilities and insufficient funds.
- Unconcerned behaviour among staff members.
- Unwillingness of those who generate waste to pay service providers.
- The presence of “sophisticated equipment” without sufficient skills to operate such equipment.
- Corruption and mismanagement of funds.
- “Civil society” is usually not involved in the decision-making arrangements.

It is evident that the collection, handling, and refurbishing of WEEE is carried out by informal recyclers, who are largely illiterate, untrained, and without experience [78]. They are usually “undocumented business” persons, usually lack training and skills, and roam the streets and waste dumps with their handcarts to collect, or in rare cases, buy abandoned WEEE and other metal scraps, which contain important elements such as iron, brass, copper, aluminium, etc. Ohajinwa [79] explains that the recycling of most of the WEEE that is generated is carried out in an informal/unsafe way, such that toxic elements are released into the environment. Mihai et al. [8] report that informal WEEE recycling practices appear to dominate the Nigerian WEEE market. These informal recycling activities often happen in backyards or small workshops in Nigerian cities (Port Harcourt), where primary methods of manual disassembly and open burning are practiced. Primitive techniques such as manual dismantling, the melting of metals, acid dipping, and open burning are often utilised to recover valuable materials from WEEE. The informal recycler does not adopt optimised methodologies for material recovery; for example, metals are usually recovered via heating WEEE on a hot plate or over an open flame. In some instances WEEE is shredded mechanically to help recover valuable metals [80,81,82,83].

Metals can be recovered from depleted lithium-ion batteries using environmentally friendly techniques [84]. Nonferrous metals can be recovered from PCBs [85]. Plastic waste such as brominated resin can be recovered from WEEE using infrared heating [86]. WEEE is recycled by removing components or valuable materials, including integrated circuits (ICs), plastics, condensers, cathode ray tubes (CRTs), printed wiring boards (PWBs), and metals, which can be reprocessed directly as reusable components for raw materials [87]. The commonly practiced process of extracting these materials is through an open burning system, which is injurious to human health and the environment [82]. The actors in the informal sectors are cart pushers, scavengers (those who sort and recover materials that can be reused or recycled), resource merchants, and recyclers [57]. Despite the problems associated with the informal WEEE management system, it serves as a source of employment and a means of livelihood for many individuals [88].

The importance of recycling is not based on the proper treatment of WEEE but on the maximum recovery of valuables in discarded WEEE from dumpsites. The crude methods employed by informal recyclers cannot adequately remove the potentially toxic elements (PTEs) in WEEE [89]. Thus, funding by all stakeholders for the upgrading of recycling infrastructure and proper integration of the recycling and waste sectors in Nigeria is a necessity. However, studies have revealed that informal recycling methods and activities, such as dismantling, open burning of plastics and wire, and indiscriminate disposal, lead to a significant level of potentially toxic elements and persistent organic pollutant emissions in air, soil, and water. The existing pathways for soil pollution and the impact on humans are described in the following sections, detailing the methods and activities of WEEE management in the informal sector [43,90].

Studies reveal that informal recycling activities, such as the open burning of wires and plastics, dismantling, and unregulated disposal, result in the release of significant levels of heavy metals as well as persistent organic pollutants into the soil, air, and underground as well as surface water [89,91]. However, a study carried out by Ezeudu and Ezeudu [91] shows that Nigeria does not have the capacity for formal recycling methods, in part due to the lack of modern recycling facilities in the country. Conversely, Omokaro [74] (p. 18) explains that the effort made to commence formal recycling in Nigeria failed due to “consumption habits” as well as the political, social, economic, and cultural context, which necessitates the services of the informal recyclers. The management of WEEE in Nigeria is faced with numerous challenges, as identified by Nnorom and Odeyingbo [92], including the following:

(a) The influx of used electrical and electronic equipment approaching their end of life is frequently combined with WEEE.
(b) The rate of collection of end-of-life electrical and electronic equipment is poor, as owners usually keep them inside their cabinets and drawers, after which they are disposed of in the general waste stream.
(c) The majority of the population appears to be ignorant of the “toxicity or hazardous nature” of WEEE.
(d) WEEE is usually disposed of alongside other waste using the same bin and is taken to the open dump, thus necessitating/promoting sorting, scavenging, etc.
(e) The flow of electronic waste via recyclers in the informal sector is more than that of formal recyclers, who are fewer in number.
(f) It is difficult to source funds to establish profitable formal WEEE recycling practices.
(g) There is weak enforcement of WEEE legislation.
(h) There is “non-implementation” of the extended producer responsibility (EPR) segment of WEEE regulations.
(i) There is a lack of appropriate infrastructure for WEEE management.
(j) The primitive techniques used by the informal recyclers give rise to environmental pollution as well as energy and resource waste.

### 1.5. Challenges of Informal Recycling in Port Harcourt, Nigeria

Goel [55] puts forward that WEEE gathering or collection in Port Harcourt is predominantly carried out by informal recyclers. They are not just involved in WEEE management alone; they gather a variety of wastes at the same time to enable them to make a living from their activities, and thus it is difficult to ascertain who is uniquely responsible for WEEE management. In [93], several informal WEEE recyclers “remain unaware of” some of the products or materials that they could recycle or recover. The same research effort explains that some of the problems of informal recycling are that it exposes human health and the environment to risk, in addition to the fact that its treatment is expensive and complex. In Vaccari et al. [94] the “spread of pollutants” from the facilities used by informal recyclers in Port Harcourt tends to affect humans via pollution transportation mechanisms “and exposure pathways”.

In Yu et al. [95] informal WEEE recycling poses many challenges, yet its practice is difficult to stop due to factors such as the increasing demand for second-hand electronics and second-hand parts (for local industries), as well as the increasing need for equipment/materials (as a result of expansion of the manufacturing industries); this is the case with Port Harcourt. The recycling of lead–acid batteries by the informal sector during dismantling results in hazardous emissions. This can affect the environment as well as the health of individuals [56,96].

In [97] the disposal of WEEE puts the environment and human life at great risk as a result of them being “exposed to” chemicals that are hazardous while the electronic components are being dismantled. The recyclers in the informal sector in Port Harcourt utilise crude recycling procedures as they lack the required infrastructure to ensure the safety of the environment and human life [95].

The informal recycling activities in Rumukurushi in Port Harcourt city are characterised by the following: the workers are predominantly young individuals, the working hours are about 9–12 h daily, and those living around the location where open burning is carried out usually experience health-related challenges, as specified in [77]. In [74] the WEEE scrappers are usually faced with the problems of bad roads, roads flooded with water after a rainfall, and the rising price of petrol, all of which affect “the availability of transport”. Awasthi et al. [98] emphasised that the majority of “developing countries” are faced with the challenges associated with recycling WEEE using the informal approach, and this is because of the large population of jobless individuals who now collect and recycle WEEE at family-owned workshops.

The informal WEEE recyclers are faced with some limitations, which can be minimised with the intervention of the government and private individuals. These limitations are specified in Alabi and Wohlmuth [57] and include:

(a) A lack of essential facilities that can enhance their efforts and activities.
(b) No monetary aid nor recognition from the government.
(c) A lack of safety equipment to protect them from the health-related risks usually associated with hazardous elements.
(d) A general absence of essential training on how to protect themselves, WEEE handling, environmental issues, etc.
(e) Lack of access to adequate orientation, training, health facilities, as well as first-aid treatment in situations of emergency.
(f) Informal recyclers do not have access to proper medical services.

Omokaro [74] suggests that recycling carried out in the informal sector gives rise to several “negative environmental effects” and hence should be stopped.

### 1.6. Factors That Affect the WEEE Management System in Port Harcourt, Rivers State, Nigeria

The factors that affect the strategies adopted to manage WEEE in Port Harcourt are specified in Okorhi et al. [56] and include:

- A lack of standardised local recycling systems.
- Incorrect identification of WEEE at the entry point.
- Lack of awareness of the toxicity of WEEE and the difficulty in differentiating near-end-of-life EEE from WEEE.
- Take-back programmes are difficult to initiate and pursue, “co-loading of near E.o.L”, second-hand vehicles with WEEE and EEE, and a lack of localised statistics on EEE and WEEE.
- The inability of the government to proffer a longstanding and feasible approach to WEEE management.
- Users of EEE have a tendency to purchase considerably “used” ones that are close to losing their useful life as opposed to ones that are new.

**Figure 3:**
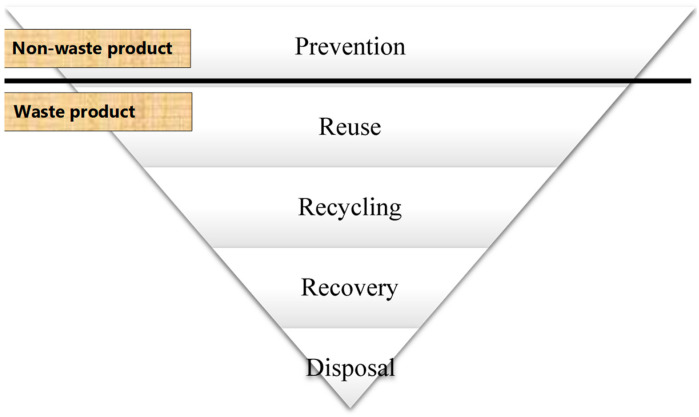
Hierarchy of waste.

Cole et al. [42] suggested that it is advisable to consider a five-point waste hierarchy for WEEE management, as shown in Figure 3.

Presently, WEEE management at MTN phone villages in Port Harcourt, Nigeria, is carried out by informal recyclers who lack basic knowledge on prevention, reuse, recycling, recovery, and disposal, as shown in Figure 3. Our study looks at approaches to change the existing WEEE management practices at the location, as its focus is to prevent the harmful and primitive style of WEEE management carried out by recyclers in the informal sector in the area and find potential applications to other regions faced with similar practices. Furthermore, if the activities of informal recyclers are drastically reduced at MTN phone villages in Port Harcourt, Nigeria the associated problems with poor recycling in the informal sector will be greatly inhibited.

## 2. Materials and Methods

This research work is geared towards “prevention”, as shown in Figure 3. The research focuses on pragmatic opportunities to reduce informal recycling at MTN phone villages in Port Harcourt by reducing the involvement of informal recyclers in the management of WEEE. Two different concepts were adopted in the study, and these were:

1. Ensuring that WEEE is recovered from informal recyclers, residents, and other areas and is sent to government-approved agencies for sorting, processing, and treatment.
2. Soliciting the support of informal recyclers to engage only in WEEE collection and gathering and to be paid for these services.

A qualitative research approach [99] was adopted, as data were gathered with the help of questionnaires. The analysis of the questionnaires was achieved using graphical methods. The study participants were selected individuals from the chosen location in Port Harcourt, Nigeria. The chosen research location was a small business village, locally known as Rumukurushi’s MTN phone village. The location is very small, such that the number of informal WEEE recyclers operating in the area is estimated to be about 30 persons. The location, even though small, is faced with the challenge of indiscriminate dumping of WEEE. This study was carried out to establish a means to limit the role of recyclers in the informal sector and the associated problems. The informal recyclers who reside in the location were consulted in order to establish a clear picture of practices in the area. The participants in the study include both males and females (i.e., 22 men and 3 women). A total of 25 participants took part in the study. Male and female participation in the survey accounted for 88% and 12%, respectively. Out of the 25 informal recyclers who took part in the study, only two had attended secondary school; the remaining attended primary school only.

The study was given ethical approval after a review of the study design and documentation by the School of Computing, Engineering & Physical Sciences Ethics Committee, University of the West of Scotland.

## 3. Results and Discussion

This study attempts to identify the main reason why the individuals in the chosen research location engage in informal WEEE recycling. The options made available to the recyclers were no job availability, zero tax payment, extra income generation, and others, as shown in Table 5.

**Figure 4:**
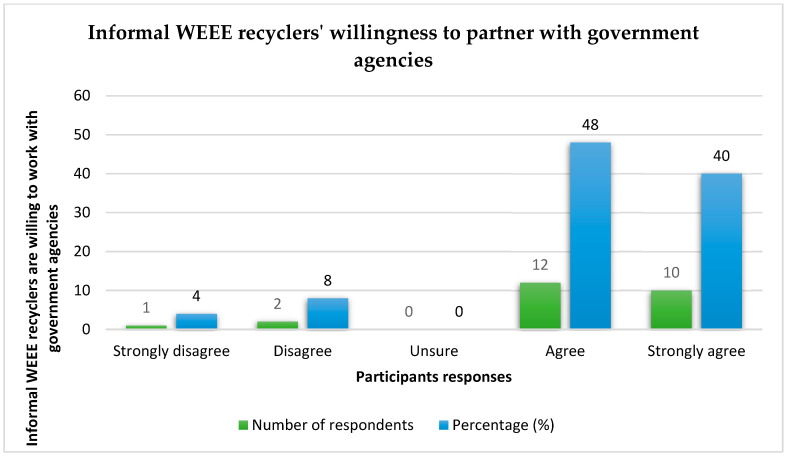
A plot of informal WEEE recyclers’ willingness to partner with government agencies.

The outcomes show that the majority of the informal recyclers (64%) admitted that the unavailability of jobs was the key reason for participating in WEEE activities. An effort was made to ascertain if the informal recyclers intend to partner with government agencies for effective WEEE management if given the opportunity. The results show that 4% of the participants strongly disagree with a partnership with government agencies, 8% disagree, 48% agree, and 40% strongly agree. The number of participants who showed positive interest in partnering with government agencies exceeds those that are not in support by a great margin. See details in Figure 4.

**Figure 5:**
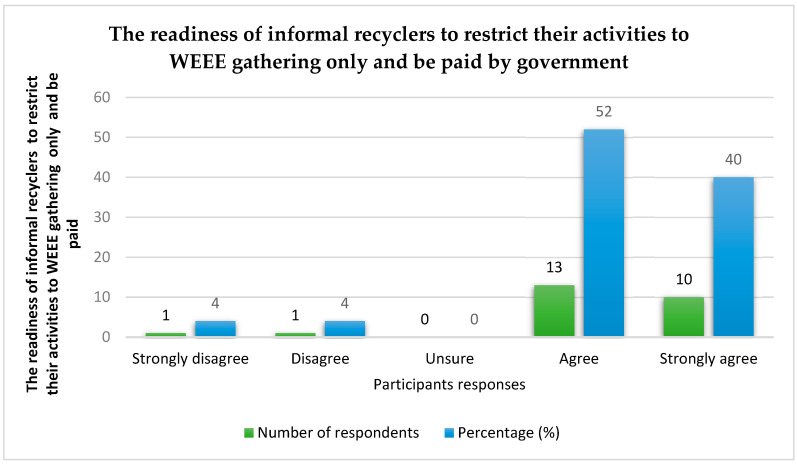
A plot of the readiness of informal recyclers to restrict their activities to WEEE gathering only and be paid.

The readiness of the recyclers in the informal sector to accept pay for the collection or gathering of WEEE and to disengage themselves from other activities (e.g., burning, treatment, etc.) in the management of WEEE was carefully examined. According to the outcome, 4% of respondents disagree and 4% strongly disagree. Furthermore, 52% and 40% agree and strongly agree, respectively. This demonstrates the readiness of informal recyclers to restrict their activities to WEEE gathering or collection if an opportunity to be paid by the government is offered to them. See details in Figure 5.

### Advantages of the WEEE Management Approach in MTN Phone Villages, Rumukurushi, a Small Settlement in Port Harcourt, Rivers State, Nigeria

The advantages associated with this approach of managing WEEE are:

- The enhancement of sustainable and safe disposal methods for materials that can cause hazards.
- The creation of room for the recovery of important elements, such as copper, gold, etc., for recycling and reuse.
- The separation of materials that can cause hazards from those that do not during WEEE dismantling is enhanced.
- With this concept, the activities of recyclers in the informal sector are easily checked and curtailed.
- WEEE repurposing, recycling, reuse, reduction, and recovery can be achieved easily.
- It has the potential to reduce the volume of tasks carried out by informal recyclers.
- It will serve as a means of motivation for recyclers in the informal sector.
- It can offer policymakers valuable insights with regard to the management of WEEE.
- With this approach in place, it is possible to minimise the hazards presented by recycling in the informal sector.
- It creates awareness of the importance of engaging experienced and well-trained workers to manage WEEE.

## 4. Conclusions

This study carried out a review of past publications on the management of WEEE in order to reduce risks to human health from activities in the informal recycling sector in MTN phone village located in Rumukurushi, a small settlement in Port Harcourt, Rivers State, Nigeria. A new concept of managing WEEE was introduced for Rumukurushi, which limits the activities of informal recyclers to WEEE collection only, in return for formal payment for their services. In this case, the informal recyclers do not take part in WEEE treatment, dismantling, refining, disposal, etc. Trained workers and government-approved offices are solely responsible for the management of WEEE. This provides increased productivity, efficiency, and safety. The study outcomes show that 48% agree to partner with government agencies, while 40% of those remaining strongly agree to the collaboration. The number of participants who showed a positive interest in partnering with government agencies exceeds that of those who were not in support by a great margin. In addition, 52% and 40% agree and strongly agree, respectively, to limit their activities exclusively to WEEE collection if the government is willing to pay for their services.

## Figures and Tables

**Table 1:** Top ten global generators of WEEE, in total mass and per capita, and the presence of national regulations, 2014.

SN	Country	Mass (kilo tons)	National Regulation	Per Capita (tons)
1	US	7072	No	22.1
2	China	6033	Yes	4.4
3	Japan	2200	Yes	17.3
4	Germany	1769	Yes	21.6
5	India	1641	No	1.3
6	UK	1511	Yes	23.5
7	France	1419	Yes	22.1
8	Brazil	1412	No	7.0
9	Russia	1231	No	8.7
10	Italy	1077	Yes	17.6

**Table 2:** The generators of WEEE and its collection per continent.

Indicators	Oceania	Europe	Asia	America	Africa
Countries within the region	13	40	49	35	53
Population within the region (millions)	39	738	4364	977	1174
Water gauge, WG (kg/inh)	17.3	16.6	4.2	11.6	1.9
Indication WG (Mt)	0.7	12.3	18.2	11.3	2.2
WEEE documented to be collected and recycled (Mt)	0.04	4.3	2.7	1.9	0.004
Rate of WEEE collection within the region	6%	35%	15%	17%	0%

**Table 3:** Typical WEEE components and their adverse health effects.

E-Waste Component	Adverse Health Effects	Electronic & Electrical Appliances with These Components
Sulphur	Throat & eye irritation, Liver, Kidney and heart damage	Lead-acid batteries
Arsenic	It has negative effect on Liver, skin, respiratory and nervous system	Phones, Microchips
Carcinogenic powder	Skin irritation, cancer	Ink Cartridges/toner
Brominated Flame retardants	Brain damage, thyroid and liver problems	Most electronic plastics
Cadmium	Neuromotor deficit in children, severe damage to kidney and lungs	Phone battery, Nickel-Cadmium rechargeable lamps
Mercury	Kidney damage, dermatitis, slower growth, reduce fertility, muscle weakness, memory loss	Phones, flat screen TVs/monitors, mechanical door bells, Fluorescent tubes
Lead	Lower IQ, hyperactivity, attention deficits, behavioural disturbances, nervous system damage.	Circuit boards, lead acid batteries, CRT monitors, some PVCs

**Table 4:** Differences between informal recycling in Port Harcourt, Nigeria, and formal recycling in other countries, e.g., Mexico.

Informal Recycling	Formal Recycling
Pickers either collect WEEE or they are provided it by consumers	WEEE is gathered via a collection service, logistic service, collection campaign, or at a clean point
Components are manually separated or dismantled	Components are mechanically separated or dismantled
No recycling facilities, only primitive recycling procedures exist	Recycling facilities exist
No delivery to qualified waste managers	Delivery to qualified waste managers takes place
No pretreatment	Pretreatment takes place

**Table 5:** The reasons why informal recyclers engage in WEEE management.

S/n	Reasons for Individual Engagement in Informal Recycling	Number of Participants in Agreement	Participants in Agreement (%)
1	Unavailability of jobs	16	64
2	Zero tax payment	1	4
3	Extra income generation	3	12
4	Other	5	20

## Data Availability

Not applicable.

## Abbreviations

WEEE: Waste electrical and electronic equipment; MTN: mobile telephone network.
